# Involvement of the FOXO6 transcriptional factor in breast carcinogenesis

**DOI:** 10.18632/oncotarget.23779

**Published:** 2017-12-30

**Authors:** François Lallemand, Ambre Petitalot, Sophie Vacher, Leanne de Koning, Karim Taouis, Bernard S. Lopez, Sophie Zinn-Justin, Nicole Dalla-Venezia, Walid Chemlali, Anne Schnitzler, Rosette Lidereau, Ivan Bieche, Sandrine M. Caputo

**Affiliations:** ^1^ Service de génétique, unité de pharmacogénomique, Institut Curie, Paris, France; ^2^ Service de génétique, unité de génétique constitutionnelle, Institut Curie, Paris, France; ^3^ Département de transfert, Institut Curie, Paris, France; ^4^ CNRS UMR 8200, Gustave Roussy Cancer Institute, Université Paris-Saclay, équipe labélisée par la Ligue contre le cancer, Villejuif, France; ^5^ Laboratoire de biologie structurale et radiobiologie, IBITEC-S (CEA) and I2BC (UMR 9198, CEA, CNRS, Univ. Paris South), Gif-sur-Yvette, France; ^6^ Centre de Recherche en Cancérologie de Lyon (CRCL)/INSERM U1052-CNRS UMR5286, Lyon, France; ^7^ EA7331, Université Paris Descartes, Paris, France

**Keywords:** gynecological cancers, cervical squamous cell carcinoma, endometrial adenocarcinoma, uc.189, prognosis

## Abstract

In mammals, FOXO transcriptional factors form a family of four members (FOXO1, 3, 4, and 6) involved in the modulation proliferation, apoptosis, and carcinogenesis. The role of the FOXO family in breast cancer remains poorly elucidated. According to the cellular context and the stage of the disease, FOXOs can have opposite effects on carcinogenesis. To study the role of FOXOs in breast carcinogenesis in more detail, we examined their expression in normal tissues, breast cell lines, and a large series of breast tumours of human origin. We found a very low physiological level of *FOXO6* expression in normal adult tissues and high levels of expression in foetal brain. *FOXO* gene expressions fluctuate specifically in breast cancer cells compared to normal cells, suggesting that these genes may have different roles in breast carcinogenesis. For the first time, we have shown that, among the various *FOXO* genes, only *FOXO6* was frequently highly overexpressed in breast cell lines and tumours. We also found that inhibition of the endogenous expression of FOXO6 by a specific siRNA inhibited the growth of the human breast cell lines MDA-MB-468 and HCC-38. FACS and Western blot analysis showed that inhibition of endogenous expression of FOXO6 induced accumulation of cells in G0/G1 phase of the cell cycle, but not apoptosis. These results tend to demonstrate that the overexpression of the human *FOXO6* gene that we highlighted in the breast tumors stimulates breast carcinogenesis by activating breast cancer cell proliferation.

## INTRODUCTION

The *FOXO* genes encode the proteins of the O-subfamily belonging to the large family of forkhead transcription factors that share a highly conserved DNA-binding domain, the forkhead domain or winged-helix domain [1, 2]. In mammals, the O-subfamily is composed of four genes: *FOXO1*, *FOXO3*, *FOXO4*, and *FOXO6*, involved in the regulation of various cellular processes, such as cell cycle progression, apoptosis, metabolism, and DNA-repair. *FOXO* genes are ubiquitously expressed in varying degrees in all mouse tissues examined [3–5]. It has been shown that *FOXO6* is also expressed in mouse embryo, mainly in the brain [6]. The transcriptional activity of FOXO proteins is regulated by posttranslational modifications, such as acetylation, ubiquitination, and phosphorylation [1]. Notably, FOXOs are negatively modulated by growth factors, such as insulin, via activation of the PI3K-AKT pathway. Activation of this pathway induces phosphorylation of FOXO proteins by AKT, leading to their exclusion from the nucleus, thereby terminating their ability to induce target genes [6, 7]. Human tumours frequently harbour activating mutations in PIK3CA (or p110α, the catalytic subunit of PI3K) or inactivating mutations in PTEN (negative regulator of the PI3K-AKT pathway), leading to over-stimulation of PI3K-AKT pathway activity [8].

Because of their anti-proliferative and pro-apoptotic functions, and the fact that conditional deletion of *FOXO1*/*2*/*4* alleles in adult mouse tissues leads to the appearance of lymphoblastic thymic lymphomas and haemangiomas, FOXOs have been considered to be tumour suppressors [2]. However, various studies have described unexpected functions of FOXOs in resistance to cancer treatment and cancer promotion, suggesting a complex role of FOXOs in this disease. Overexpression of *FOXO1* and *FOXO3* has been shown to inhibit the growth of breast cancer cells [9–12]. IκB kinase and ERk promote breast carcinogenesis *via* inhibition of FOXO3 [9, 11]. Moreover, cytoplasmic FOXO3 staining is positively correlated with poor patient survival [9]. These results strongly suggest that FOXOs act as tumour suppressors in breast cancer. However, FOXO1 and 3 have also been implicated in the promotion of breast tumour cell invasion [13, 14]. The results reported by Sisci *et al.* suggest that the role of FOXO3 in breast cancer is linked to the oestrogen receptor α (ERα) status: in ERα-positive cells, FOXO3 inhibits breast carcinogenesis, while in ERα-negative cells, FOXO3 tend to promote breast carcinogenesis [15]. The role of FOXOs in breast carcinogenesis therefore appears to depend on the cellular context and the stage of disease. The possible role of other FOXOs proteins (FOXO4 and 6) in breast cancer remains unknown.

To more clearly define the role of the FOXO family in breast cancer, we studied their expression in normal tissues, breast cell lines, and tumours of human origin. Surprisingly, we found that *FOXO6*, but not *FOXO1*, *3*, and *4*, was frequently overexpressed in breast cell lines and tumours compared to normal cells, suggesting that this *FOXO* gene could act as an oncogene in human breast carcinogenesis. To further examine this possibility, we studied the effect of inhibition of endogenous FOXO6 expression on cell growth of two different human breast cell lines expressing high levels of *FOXO6*.

## RESULTS

### Expression analysis of *FOXO* genes in a variety of human normal tissues and cancer

To study the involvement of the four *FOXO* genes in cancer, *FOXO* gene expression was first determined in 24 normal human tissues by qRT-PCR (Supplementary Table 1). These genes were ubiquitously expressed in all normal tissues examined. The highest *FOXO1* expression was detected in the uterus, skeletal muscle and ovary, the highest expression *FOXO3* was detected in bone narrow and skeletal muscle, and the highest *FOXO4* expression was detected in the placenta, adrenal gland, ovary and skeletal muscle. Moderate to low *FOXO6* expression was observed in normal adult tissues and was lower than the expression of other *FOXO* genes. This *FOXO* gene was highly expressed in foetal brain (mRNA expression level = 502) but not in foetal liver (mRNA expression level = 9) in keeping with earlier report [6]. The human *FOXO6* gene is therefore expressed in a specific temporal and spatial pattern.

We then compared the expression of these genes in four types of cancer and corresponding normal tissues: breast, brain, bladder, and colon. Five normal tissues and 10 cancer tissues were analysed for each cancer type. *FOXO1*, *FOXO3*, and *FOXO4* were significantly underexpressed in tumour samples: in breast, bladder and colon tumours for *FOXO1*, in breast tumours for *FOXO3*, and in breast, bladder, and colon tumours for *FOXO4* (Supplementary Figure 1). Interestingly, in contrast with the other tumors, slight significant overexpression of *FOXO1* was observed in brain tumours, and the expression of *FOXO3* was found to be overexpressed in some of these tumors.

**Figure 1:**
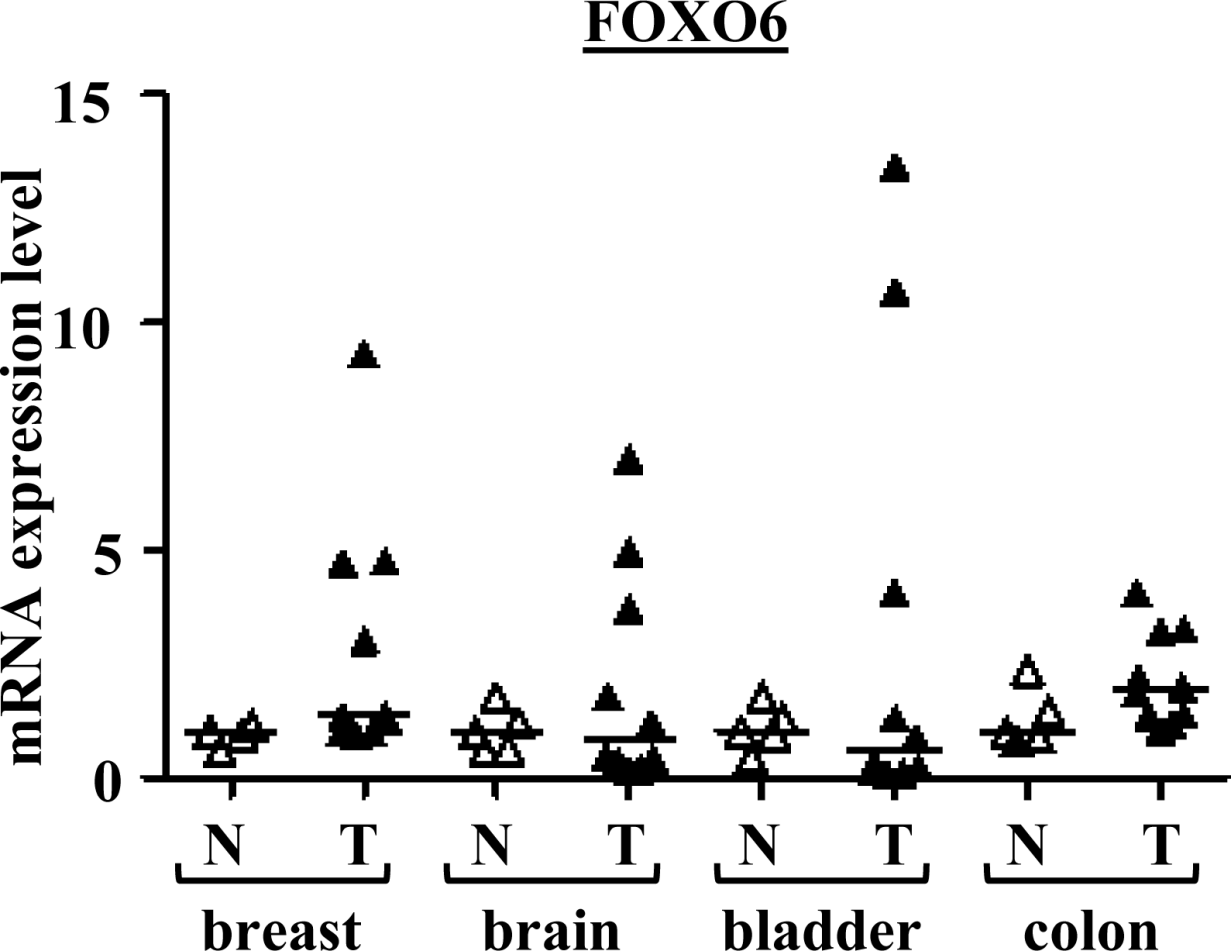
*FOXO6* mRNA expressions in various cancers and normal tissues Scatter dot plot with median of qRT-PCR data for *FOXO6* in the series of breast, brain, bladder and colon tissues (*n* = 5 normal tissues and *n* = 10 tumour tissues for each). *p*-values (Mann-Whitney *U* Test) are indicated: ^*^, 0.01 < *p*-value < 0.05; ^**^, *p*-value < 0.01.

Surprisingly, very heterogeneous *FOXO6* expression was observed in the various cancer tissues examined (Figure 1). Most importantly, this gene was found to be highly overexpressed in several bladder, brain and breast tumours. Therefore, FOXO6 would play an important role in carcinogenesis, notably in breast cancer. These observations have led us to study further the involvement of this gene in breast carcinogenesis.

### Expression analysis of *FOXO* genes in human breast cell lines and tumours

To study further the role of *FOXO6* in breast cancer, first we used qRT-PCR to examine the expression of *FOXO* genes in a series of 39 human breast cell lines (including seven normal breast cell lines (N) and thirty-two tumorigenic breast cell lines (T), Supplementary Table 2) and in a large series of 527 human breast tumours (clinical parameters presented in Supplementary Table 3). We confirmed that *FOXO6*, but not *FOXO1*, *3* or *4*, was frequently overexpressed in breast cell lines (25%) and in breast tumours (26.9%) (Table 1 and Supplementary Table 2).

**Table 1:** mRNA expressions of *FOXO* in breast cell lines and tumors

Cell line/ Tumor	Gene	*FOXO1*	*FOXO3*	*FOXO4*	*FOXO6*
	**Expression**	**Number (%)**			
Tumorigenic cell lines (*n* = 32)	Overexpression Normal expression	0 (0) 32 (100)	0 (0) 32 (100)	0 (0) 32 (100)	8 (25) 24 (75)
Tumors (*n* = 527)	Overexpression Normal expression	1 (0.2) 526 (99.8)	23 (4.4) 504 (95.6)	6 (1.1) 521 (98.9)	142 (26.9) 385 (73.1)

*FOXO6*-expression values of the breast cell lines were normalized so that the ‘basal *FOXO6* mRNA level’ (smallest quantifiable amount of mRNA (Ct = 35)) was equal to 1. Overexpression was defined as Ct values under 30 (values above 32 (2^ΔCt^ = 2^35–30^ = 32)).

*FOXO6*-expression values of the breast tumors were normalized so that the median *FOXO6*-expression value of normal breast tissues was equal to 1. Overexpression was defined as threefold variations of expression relative to the median expression of normal samples.

**Figure 2:**
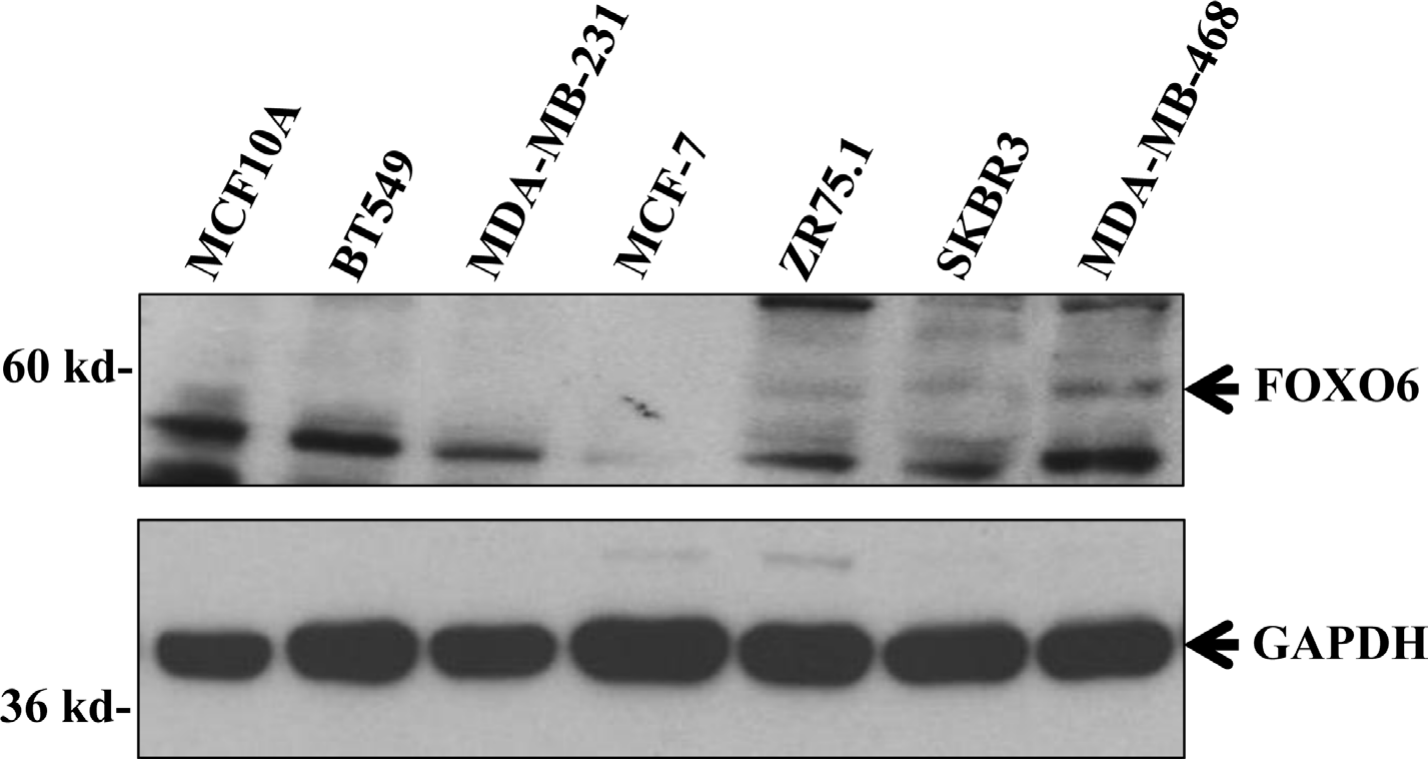
FOXO6 protein expression in various breast cell lines Cellular extracts of various breast cell lines were analysed by immunoblotting for their expressions of FOXO6 and GAPDH (loading control).

Western blot analysis demonstrated FOXO6 protein expression in breast cell lines expressing a high level of *FOXO6* mRNA, but not in breast cell lines expressing low levels of this mRNA or in the normal mammary cell line MCF10A, confirming FOXO6 protein overexpression in breast cancer cells (Figure 2 and Supplementary Table 2).

Altogether, these results indicate that, among the four members of the *FOXO* gene family, only *FOXO6* is overexpressed in breast cancer.

### *FOXO6* expression is negatively correlated with expression of an active form of AKT1 and positively correlated with PTEN expression in human breast tumours

The PI3K-AKT pathway has been shown to inhibit *FOXO1*, *3* and *4* expression [16], suggesting that *FOXO6* overexpression in breast cancers could be due to impaired activity of this pathway. To test this hypothesis, we investigated the correlations between *FOXO6* mRNA expression level and various proteins involved in the PI3K-AKT pathway.

The levels of fourteen proteins (non-phosphorylated and/or phosphorylated) involved in the PI3K-AKT pathway were analysed using RPPA assays in 224 samples from our series of 527 human breast tumours (Table 2). A negative correlation was observed between the expressions of *FOXO6* and AKT1 phosphorylated on serine 473, a marker of PI3K-AKT pathway activity [17] (Spearman test: *r* = –0.192, *p* = 0.0039). A positive correlation was also observed between the expressions of FOXO6 and PTEN, a negative regulator of the PI3K-AKT pathway [17] (Spearman test: *r* = +0.135, *p* = 0.044, confirmed at the mRNA level: *r* = +0.212, *p* = 0.000018).

**Table 2:** Relationship between levels of *FOXO6* mRNA and a panel of proteins of the PI3K-AKT pathway in a series of 224 breast tumors

Proteins of the PI3K-AKT pathway	r^a^	*p*-value^a^
PTEN	**+0.135**	**0.044**
INPP4b	+0.036	NS
Akt1	+0.060	NS
p-Akt1.ser473	**–0.192**	**0.0039**
Akt2	**–**0.010	NS
mTor	**–**0.100	NS
p-mTor.ser2448	**–**0.019	NS
FOXO1	**–**0.086	NS
TSC2	+0.038	NS
p70.S6.Kinase	+0.076	NS
p-p70.S6.Kinase.thr389	+0.014	NS
S6.Ribosomal.protein	+0.019	NS
p-S6.Ribosomal.protein.ser235/ser236	**–**0.058	NS
p-S6.Ribosomal.protein.ser24	**–**0.046	NS

a:Spearman rank correlation Test. In bold: *p*-values < 0.05.

These observations indicate that the FOXO6 overexpression observed in human breast cancers is correlated with a low PI3K-AKT pathway activity.

### Relationship between *FOXO6* mRNA expression in human breast tumours and classical clinicopathological parameters

We investigated the relationship between *FOXO6* expression and clinicopathological parameters (Table 3). No correlation was observed between *FOXO6* mRNA levels and age, grade, lymph node status, macroscopic tumour sizes, molecular subtypes and presence of metastases. However, a weakly positive correlation was observed between *FOXO6* mRNA overexpression and PR-positive status (*p* = 0.035) and, more interestingly, with the proliferation marker *MKI67* (*p* = 0.027).

**Table 3:** Relationship between *FOXO6* transcript level and classical biological parameters in a series of 527 breast tumors

Clinical biological parameters		Number of patients (%)		*p*-value^a^
	Total population	*FOXO6* mRNA expression ˂ 3 relative to normal	*FOXO6* mRNA expression ≥ 3 relative to normal	
Total	527 (100)	385 (73.1)	142 (26.9)	
Age ≤50 >50	125 (23.7) 402 (76.3)	99 (79.2) 286 (71.1)	26 (20.8) 116 (28.9)	0.076 (NS)
SBR histological grade^b,c^ I II III	60 (11.7) 241 (47.1) 211 (41.2)	42 (70) 187 (77.6) 145 (68.7)	18 (30) 54 (22.4) 66 (31.3)	0.088 (NS)
Lymph node status^d^ 0 1–3 >3	159 (30.5) 250 (47.9) 113 (21.6)	114 (71.7) 179 (71.6) 88 (77.9)	45 (28.3) 71 (28.4) 25 (22.1)	0.42 (NS)
Macroscopic tumor size^e^ ≤25 mm >25 mm	248 (48) 269 (52)	181(73) 197 (73.2)	67 (27) 72 (26.8)	0.95 (NS)
ERα status Negative Positive	181 (34.3) 346 (65.7)	140 (77.3) 245 (70.8)	41 (22.7) 101 (29.2)	0.11 (NS)
PR status Negative Positive	255 (48.4) 272 (51.6)	197 (77.2) 188 (69.1)	58 (22.7) 84 (30.9)	**0.035**
ERBB2 status Negative Positive	397 (75.3) 130 (24.7)	297 (74.8) 88 (67.7)	100 (25.2) 42 (32.3)	0.11 (NS)
Molecular subtypes HR– ERBB2– HR– ERBB2+ HR+ ERBB2– HR+ ERBB2+	102 (19.4) 72 (13.7) 295 (56) 58 (11)	82 (80.4) 52 (72.2) 215 (72.9) 36 (62.1)	20 (19.6) 20 (27.8) 80 (27.1) 22 (37.9)	0.093 (NS)
Histological types^f^ Ductal Lobular Other	398 (89.6) 28 (6.3) 18 (4.1)	294 (73.9) 20 (71.4) 14 (77.8)	104 (26.1) 8 (28.6) 4 (22.2)	0.89 (NS)
MKI67 mRNA expression^h^ Median (range)	12.5 (0.8–313)	11.91 (0.8–313)	14.05 (1.74–117.3)	**0.027** i
Metastasis No Yes	317 (60.2) 210 (39.8)	229 (72.2) 156 (74.3)	88 (27.8) 54 (25.7)	0.60 (NS)

a: Chi-squared Test; b: Scarff Bloom Richardson classification; c: information available for 512 patients; d: information available for 522 patients; e: information available for 517 patients; f: information available for 444 patients; h: information available for 438 patients; i: Mann-Whitney’s *U* test. Abbreviations: NS: not significant; ERα: estrogen receptor alpha; PR: progesteron receptor; ERBB2: human epidermal growth factor receptor 2; HR: hormone receptor.

**Figure 3:**
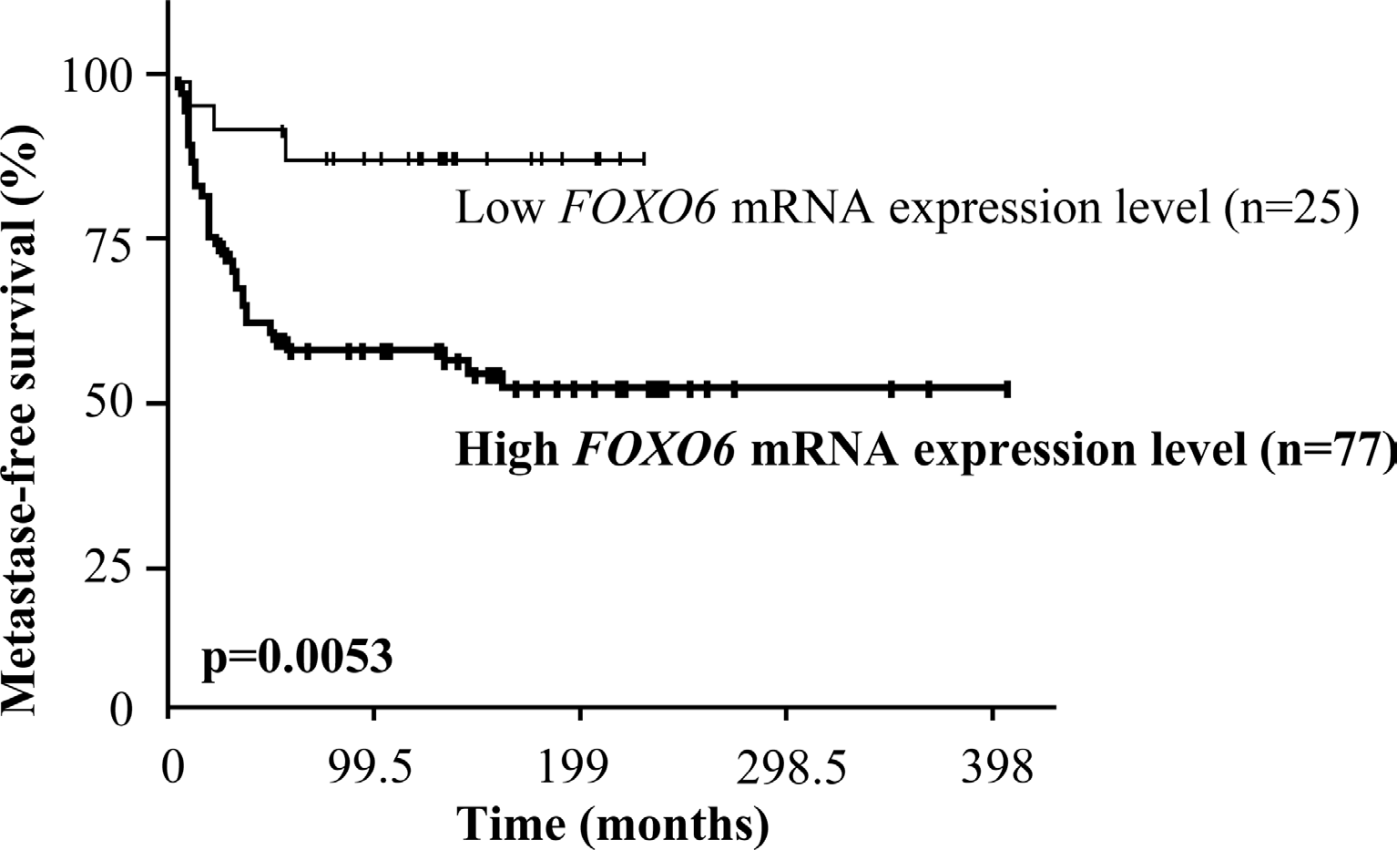
Survival curves of two groups of patients according to *FOXO6* mRNA expression level in the cohort of 102 triple negative breast tumors AUC analysis was used to divide the population into two relevant *FOXO6* expression subgroups.

Most importantly, a log rank test was used to identify relationships between metastase-free survival (MFS) and *FOXO6* mRNA levels. *FOXO6* overexpression was not a prognostic marker in our series of 527 breast tumors (data not shown). We therefore investigated if *FOXO6* overexpression could be a prognostic marker in a subpopulation of breast cancer (see Table 3). The high expression of *FOXO6* was not a marker of poor prognostic in HR- ERBB2+, HR+ ERBB2-, HR+ ERBB2+, lobular, or ductal breast cancer. However, this approach allowed us to highlight that the high expression of *FOXO6* was a marker of poor prognostic in our subpopulation of breast tumors HR- and ERBB2- (triple negative breast tumors) (*p =* 0.0053, Figure 3). The classical biological parameters: age, SBR histological grade, lymph node status, macroscopic tumor size, and PIK3CA mutation status, were not prognostic markers in this series of triple negative breast tumors (data not shown). The prognostic value of the *FOXO6* expression in this subpopulation of breast tumors is therefore independent of biological parameters studied.

Altogether, our results raise the hypothesis that FOXO6 overexpression may play an important role in the development of triple negative breast tumor.

### Effect of *FOXO6* siRNA on the growth of two different human breast cell lines

**Figure 4:**
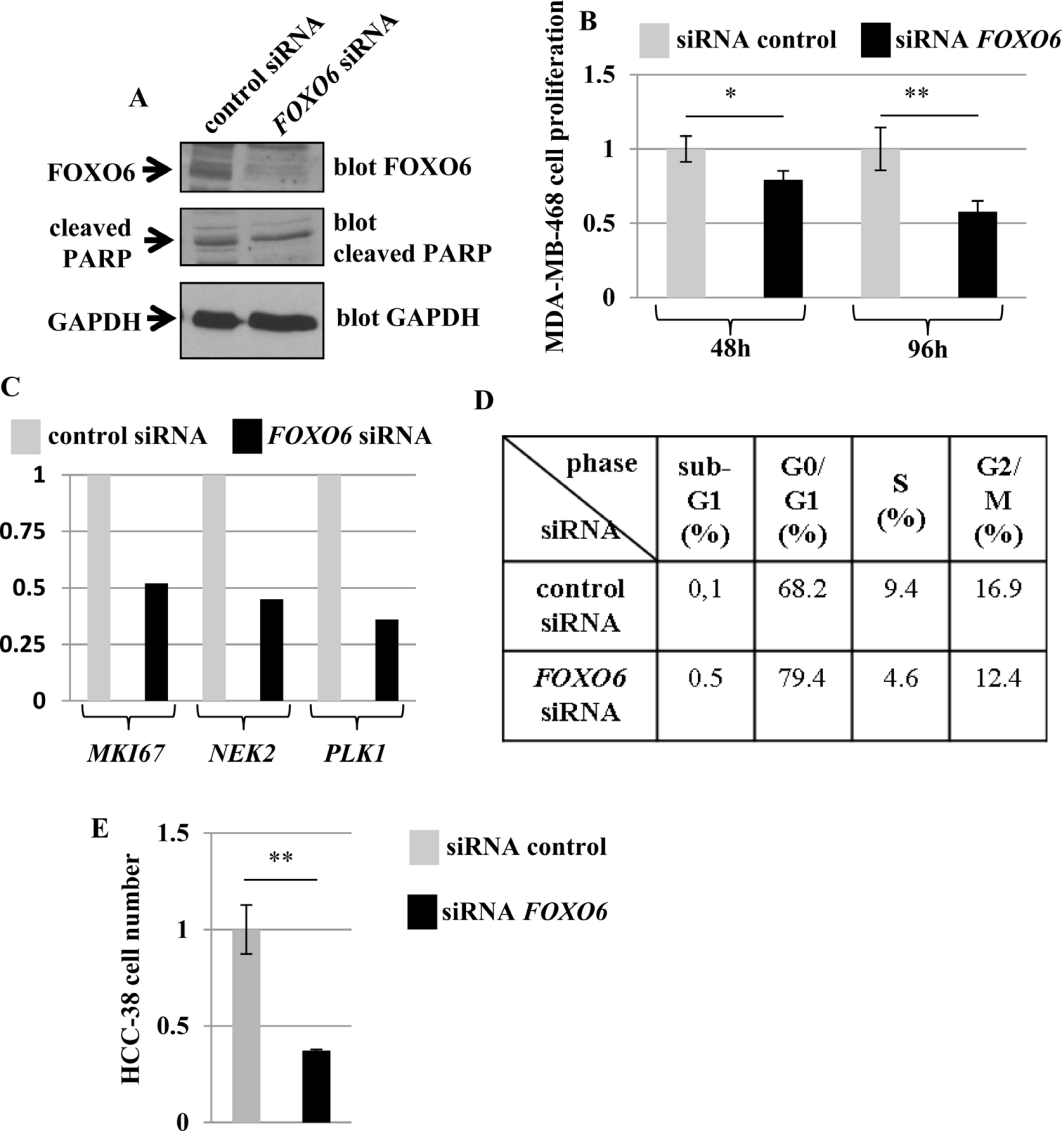
Effect of a specific *FOXO6* siRNA on the growth of the human breast cell lines MDA-MB-468 and HCC-38 **(A)** Effect of *FOXO6* siRNA on the expressions of FOXO6 and cleaved PARP (marker of apoptosis). MDA-MB-468 cells were transfected with control siRNA or *FOXO6* siRNA as indicated. Six days after transfection, the expressions of FOXO6, cleaved PARP, and GAPDH (loading control) were determined by Western blot. **(B**–**D)** Effect of *FOXO6* siRNA on cell growth. MDA-MB-468 cells were transfected with control siRNA or *FOXO6* siRNA as indicated. The viable cell count was determined two and four days after transfection (B). Six days after transfection, the expressions of *MKI67*, *NEK2*, and *PLK1* were evaluated by qRT-PCR (expressions were normalised to that detected in cells transfected with control siRNA) (C) and FACS analysis was performed (D). **(E)** Effect of *FOXO6* siRNA on cell growth of HCC-38 cell line. Cells were transfected with control siRNA or *FOXO6* siRNA as indicated. The viable cell count was determined 7 days after transfection.

To further investigate the possibility that FOXO6 might modulate the proliferation of breast cancer cells, we examined the effect of inhibiting the expression of endogenous FOXO6 on proliferation of the breast cell line MDA-MB-468 expressing high levels of this *FOXO* gene (Supplementary Table 2 and Figure 2). A specific *FOXO6* siRNA with confirmed efficacy and specificity was used to inhibit FOXO6 expression in our cellular model (Figure 4A and Supplementary Figure 2).

Inhibition of FOXO6 expression in MDA-MB-468 cells resulted in inhibition of cell proliferation (Figure 4B). In order to confirm this observation, three genes involved in proliferation (*MKI67, NEK2*, and *PLK1* [18]) were quantified by qRT-PCR in MDA-MB-468 cells treated with control siRNA or *FOXO6* siRNA. *MKI67, NEK2* and *PLK1* expressions were 1.9-, 2.2-, and 2.8-fold lower, respectively, in MDA-MB-468 cells treated with *FOXO6* siRNA compared to control cells (Figure 4C). The *FOXO6* siRNA also induced a positive effect on expression of the *PPARGC1A* gene, an established target gene of FOXO6 [4], confirming the specificity of this siRNA.

FACS analysis showed that *FOXO6* siRNA treatment decreased the number of cells located in the S and G2/M phases of the cell cycle, induced an accumulation of cells in the G0/G1 phase, and did not increase the number of cells with sub-G1 DNA content (apoptotic cells) (Figure 4D). Moreover, *FOXO6* siRNA had no effect on the expression of cleaved PARP, a marker of apoptosis (Figure 4A).

These results strongly suggest that inhibition of FOXO6 expression affects the growth of MDA-MB-468 cells mainly by inducing cell cycle arrest in the G0/G1 phase.

To validate our results, we also tested the effect of the siRNA *FOXO6* on proliferation of the breast cell line HCC-38, another breast cell line expressing high levels of *FOXO6* gene (Supplementary Table 2). The siRNA *FOXO6* inhibited, as for the MDA-MB-468 cells, the cell proliferation (Figure 4E).

## DISCUSSION

This study demonstrates specific fluctuations of *FOXO* gene expressions in human breast cancer, suggesting that these genes play different roles in this cancer. We showed that *FOXO1, 3* and *4* are frequently underexpressed these genes could therefore act as tumour suppressor genes. This hypothesis is supported by the work of Guttilla and White (2009) showing that *FOXO1* mRNA is down-regulated in breast cancers compared to normal breast tissue, and that overexpression of *FOXO1* induces MCF-7 cell death [12]. Many observations indicate that *FOXO3* also exerts tumour suppressor activity in breast cancer [2]. However, alteration of *FOXO3* expression does not appear to be a major mechanism of inhibition of the biological function of FOXO3 in this cancer type, as no significant variation of the expression of this gene was observed in our large series of breast cancers.

Surprisingly, we found that *FOXO6* was often highly overexpressed in human breast tumours and cell lines at both the mRNA and protein levels. In contrast with *FOXO1*, *FOXO3*, and *FOXO4*, *FOXO6* could therefore be an oncogene in breast cancer. This finding is consistent with the fact that *FOXO6* is the most distant member of the FOXO family [6]. Indeed, FOXO6 exhibits major structural differences compared to the other three family members, and, unlike FOXO1 and 3, activation of the PI3K-AKT pathway by growth factors inhibits FOXO6 transcriptional activity mainly via a mechanism independent of shuttling to the cytosol [6, 19]. FOXO1, 3, and 4 contain an N- and C-terminal AKT motif and a third AKT motif located in the forkhead domain. FOXO6 lacks the conserved C-terminal AKT motif, which is the cause of the shuttling impairment [1, 19]. However, the reason why FOXO6 plays a different role in breast cancer compared to the other FOXO members has yet to be elucidated.

To our knowledge, this is the first time that *FOXO6* overexpression has been demonstrated in breast cancer. In particular, no data concerning *FOXO6* expression in breast cancer were found in the cbioportal (www.cbioportal.org) or TCGA databases due to the absence of a specific probe for FOXO6. Low to moderate *FOXO6* expression was observed in all human adult tissues examined in this study, but high *FOXO6* expression was detected in foetal brain tissue in keeping with earlier report [20]. It is noteworthy that we observed overexpression of this *FOXO* gene also in several brain, bladder and colon tumours. *FOXO6* gene would therefore be involved in various cancer types.

The molecular mechanisms responsible for altered *FOXO6* expression in breast cancer are unknown. *FOXO6*, located at 1p34.2, is amplified in only 2% of breast cancers (www.cbioportal.org). The *FOXO6* overexpression observed in human breast tumours and cell lines would therefore not be due to this molecular mechanism. The study by Guttilla *et al.* indicated that *FOXO1* expression is modulated by several microRNAs in breast cancer cells [12]. However, the involvement of microRNAs in the regulation of *FOXO6* expression has not been described [2]. We found a negative correlation between the expressions of *FOXO6* and AKT phosphorylated on serine 473, a marker of the PI3K-AKT pathway activity, and a positive correlation between the expressions of FOXO6 and PTEN at the mRNA and protein level, a negative regulator of the PI3K-AKT pathway [17]. *FOXO6* overexpression is therefore associated with low activity of the PI3K/AKT pathway in breast cancers. Essaghir *et al.* showed that activation of the PI3K/AKT pathway by various growth factors inhibited the expression of *FOXO1*, *FOXO3*, and *FOXO4* in human fibroblasts [16]. These observations and our results therefore suggest that the *FOXO6* overexpression that we have highlighted in human breast cancers could be at least partly due to low activity of the PI3K/AKT pathway. Other studies are required to more clearly define the molecular mechanisms responsible for FOXO6 overexpression in breast cancer.

We found that a specific siRNA *FOXO6* inhibited growth of two different breast cell lines expressing high levels of *FOXO6* gene. FACS and Western blot analysis showed that inhibition of endogenous FOXO6 expression induced accumulation of MDA-MB-468 cells in G0/G1 phase of the cell cycle, but did not induce apoptosis. Our results therefore strongly suggest that *FOXO6* might be an oncogene in human breast cancer, which positively regulates cell proliferation by activating the progression of cancer cells through the G0/G1 phase. This hypothesis is supported by the results of various studies. Li Qinyu *et al.* (2013) showed that *FOXO6* mRNA and protein levels are upregulated in gastric cancer tissues, and this overexpression promotes gastric cancer cell tumorigenicity *via* upregulation of Myc [21]. However, we have not found any correlation between the expressions of *FOXO6* and *c-Myc* in breast tumors (data not shown), strongly suggesting that the overexpression of FOXO6 would act on breast carcinogenesis *via* a specific mechanism independent of the transcriptional factor Myc. Moreover, Chen *et al.* demonstrated that *FOXO6* is overexpressed in hepatocellular cancer and that *FOXO6* siRNA increases the percentage of cells in G0/G1 phase [22]. However, *FOXO6* has also been shown to be downregulated in lung cancer compared to adjacent normal tissue [23]. Moreover, *FOXO6* overexpression inhibits the proliferation of A549 human lung cancer cells, whereas knockdown of endogenous *FOXO6* expression enhances cell proliferation [23]. *FOXO6* therefore behaves like a tumour suppressor gene in lung cancer. These findings suggest that FOXO6 may have opposite roles in cancer depending on the cancer type.

In conclusion, we provide evidences strongly suggesting that the overexpression of FOXO6 promotes breast carcinogenesis by stimulating cellular proliferation. *FOXO6*, which is intensely expressed in breast cancers, but expressed at very low levels in normal adult tissues, may therefore be a potential candidate as a target for breast cancer therapy.

## MATERIALS AND METHODS

### Patients and samples

Samples of 527 primary unilateral invasive breast tumours (composed of 89.6% of ductal breast cancer, 6.3% of lobular breast cancer, and 4.1% for the other subtypes from information available for 444 patients) excised from women managed at Institut Curie-René Huguenin Hospital (Saint-Cloud, France) from 1978 to 2008 were analysed. Samples were immediately stored in liquid nitrogen until mRNA and protein extraction. Tumour samples were considered suitable for our study when the proportion of tumour cells exceeded 70%. All patients (mean age: 62 years, range: 29–91 years) met the following criteria: primary unilateral non metastatic breast carcinoma, for which complete clinical, histological and laboratory data were available; no neoadjuvant radiotherapy or chemotherapy; and complete follow-up at Institut Curie-René Huguenin Hospital. Treatment consisted of modified radical mastectomy in 320 cases (61.1%) and breast-conserving surgery plus locoregional radiotherapy in 204 cases (38.9%) (information available for 524 patients). Patients underwent physical examination and routine chest radiography every 3 months for 2 years, then annually. Mammograms were performed annually. Adjuvant therapy was administered to 415 patients, consisting of chemotherapy alone in 129 cases, hormone therapy alone in 178 cases and both treatments in 108 cases. The histological type and the number of positive axillary nodes were established at the time of surgery. The malignancy of infiltrating carcinomas was scored according to Scarff-Bloom-Richardson’s (SBR) histo-prognostic system. Hormone receptor (HR) (estrogen receptor α (ERα), progesterone receptor (PR)) and human epidermal growth factor receptor 2 (ERBB2) status were determined by protein assay using biochemical methods (dextran-coated charcoal method, enzyme immunoassay or immunohistochemistry) and confirmed by real-time quantitative RT-PCR assays [24, 25]. The population was divided into four groups according to HR (ERα and PR) and ERBB2 status, as follows: HR+/ERBB2+ (*n* = 58), HR+/ERBB2- (*n* = 295), HR-/ERBB2+ (*n* = 72) and HR-/ERBB2- (*n* = 102). The median follow-up was 9.1 years (range: 5 months to 33 years); 210 patients developed metastatic disease. Sixteen specimens of adjacent normal breast tissue from breast cancer patients or normal breast tissue from women undergoing cosmetic breast surgery were used as sources of normal mRNA.

Samples of 39 breast tissue-derived cell lines were analysed. These cell lines, obtained from the American Type Culture Collection (ATCC, Manassas, VA, USA) or the German Resource Centre for Biological Material (DSMZ, Braunschweig, Germany), were cultured under the conditions recommended by the suppliers, and authenticated in our laboratory by using the GenePrint 10 System kit (Promega, Madison, WI, USA) just before the extraction.

We also analysed mRNA samples from 24 normal adult tissues [26, 27] and four types of normal and cancer tissues: breast, brain, bladder, and colon (5 normal tissues and 10 cancer tissues were examined for each cancer).

### Ethic approval and consent to participate

All patients who entered our institution before 2007 were informed that their tumor samples might be used for scientific purposes and had the opportunity to decline. Since 2007, patients entering our institution have given their approval also by signed informed consent. This study was approved by the local ethics committee (Breast Group of René Huguenin Hospital).

### Cell culture

MDA-MB-468 and HCC-38 cells, purchased from ATCC, were maintained in DMEM or RPMI medium respectively containing 10% foetal bovine serum (Invitrogen, Carlsbad, CA) and 1% antibiotics (50 μg/mL penicillin, 50 μg/mL streptomycin, 100 μg/mL neomycin), and grown at 37°C in a humidified atmosphere of 5% (v/v) CO_2_ in air. This cell line was authenticated in our laboratory by using the GenePrint 10 System kit (Promega, Madison, WI, USA). We perform authentication of our cell lines each 20 passages.

### RNA extraction

Total RNA was extracted from normal human tissues, and breast cell lines and tumours by using acid-phenol guanidium, as previously described [28]. RNA quality was determined by electrophoresis on agarose gels, staining with SYBR^®^ Safe (ThermoFisher Scientific, San Jose, CA, USA) and visualization of the 18S and 28S RNA bands under blue light.

### Real-time qRT–PCR

Quantitative values were obtained from the cycle number (Ct value) at which the increase in the fluorescence signal associated with exponential growth of PCR products started to be detected by the laser detector of the ABI Prism 7900 sequence detection system (Perkin Elmer Applied Biosystems, Foster City, CA, USA), using the PE Biosystems analysis software (Perkin Elmer Applied Biosystems) according to the manufacturer’s manuals. As the precise amount of total mRNA added to each reaction mix (based on optical density) and its quality (i.e., lack of extensive degradation) are both difficult to assess, we therefore also quantified *TBP* gene transcripts (Genbank accession NM_003194) encoding the TATA box-binding protein (a component of the DNA-binding protein complex TFIID) as an endogenous RNA control and normalised each sample on the basis of its *TBP* content. *TBP* was selected as endogenous control due to the moderate prevalence of its transcripts and the absence of any known *TBP* retro-pseudogenes (retro-pseudogenes lead to co-amplification of contaminating genomic DNA and consequently interfere with qRT–PCR, despite the use of primers in separate exons) [24]. Results expressed as N-fold differences in *FOXO* target gene expression relative to *TBP* gene expression and termed ‘N_FOXO_’ were determined as N_FOXO_ = 2^ΔCtsample^, where the ΔCt value of the sample was determined by subtracting the Ct value of the *FOXO* gene from the Ct value of the *TBP* gene. *TBP* was then used as endogenous control. N_FOXO_ values of the samples were also subsequently normalised so that the median N_FOXO_ values for normal breast tissues (N) was equal to 1 (Tables 1 and 3, and, Figure 1, and Supplementary Figure 1), and/or the ‘basal mRNA level’ (smallest quantifiable amount of mRNA (Ct = 35)) was equal to 1 (Table 1 and Supplementary Tables 1 and 2). Primers for *TBP* and *FOXO* genes were chosen with the assistance of Oligo 6.0 software (National Biosciences, Plymouth, MN, USA) (Supplementary Table 4). We scanned the dbEST and nr databases to confirm the total gene specificity of the nucleotide sequences chosen for the primers. To avoid amplification of contaminating genomic DNA, one of the two primers was placed at the junction between two exons. Agarose gel electrophoresis was used to verify the specificity of PCR amplicons. Total RNA extraction, cDNA synthesis and PCR were performed under previously described conditions [28]. Over- and under-expressions were defined as threefold variations of expression relative to the median expression of normal samples, or as Ct values under 30 (values above 32 (2^ΔCt^ = 2^35–30^ = 32)) for normalization relative to ‘the basal mRNA level’.

### siRNA experiments

Control siRNA and *FOXO6* siRNA (1027281 and SI05195617, respectively, Qiagen, Santa Clarita, CA) were transfected using HiPerfect transfection Reagent (Qiagen, Santa Clarita, CA) according to the manufacturer’s instructions. 48 hours (Figure 2B) or 72 hours (Supplementary Figure 3, and Figure 2A, 2C, and 2D) after the transfection, cells were transfected once again.

### Western blotting

Samples analysed in this study come from breast cell lines cultured under the conditions recommended by the suppliers, and authenticated in our laboratory by using the GenePrint 10 System kit (Promega, Madison, WI, USA). We perform authentication of our cell lines each 20 passages.

Methods are described in detail elsewhere [29]. Briefly, proteins were extracted from cell culture using TNMG buffer (20 mM Tris-HCl (pH 8), 150 mM NaCl, 5 mM MgCl_2_, 10% glycerol, 0.5% NP-40, pH 8) supplemented with protease inhibitors. For the siRNA experiments, cells were seeded in p60 plates (500,000 cells per well), transfected with control siRNA or *FOXO6* siRNA and the cellular extracts were prepared six days after transfection. The following antibodies were used in this study: anti-GAPDH (sc-20357, Santa Cruz Biotechnology, Santa Cruz, CA), used as internal control, anti-FOXO6 (19122-1-AP, Proteintech, Chicago, USA), and anti-cleaved PARP (9541, Cell Signaling, Beverly, MA, USA). Proteins were detected by the ECL Western Blotting Analysis System procedure (GE Healthcare, Buckinghamshire, UK).

### Cell proliferation assay

Cells were seeded in 96-well plates (10,000 cells per well) and transfected with control siRNA or *FOXO6* siRNA. Cell proliferation was determined 48 hours and 96 hours after transfection using the Cell Titer kit (Promega, Madison, WI, USA) according to the manufacturer’s instructions. Results were expressed as mean ± s.d. of triplicates from a representative experiment.

### RPPA

RPPA was performed as previously described [30].

### FACS analysis

Cells were seeded in p60 plates (500,000 cells per well) and transfected with control siRNA or *FOXO6* siRNA. Six days after transfection, cells were harvested and DNA content was assessed by propidium iodide staining of methanol-fixed cells and monitoring by FACScan (LSRII).

### Statistical analysis

The relative expression of each gene was characterized by the median and the range. Relationships between mRNA expression of genes and clinical parameters, and target mRNA and protein were assessed by nonparametric tests, Kruskal-Wallis *H* test (relationship between one quantitative parameter and one qualitative parameter) and Spearman’s rank correlation test (relationship between two quantitative parameters). To visualize the efficacy of a molecular marker to discriminate between two populations (patients that developed/or did not develop metastases) in the absence of an arbitrary cut-off value, data were summarized in a ROC (receiver operating characteristic) curve [31].The AUC (area under the curve) was calculated as a single measure to discriminate efficacy. Survival distributions were estimated by the Kaplan-Meier method, and the significance of differences between survival rates was ascertained with the log-rank test. Metastasis-free survival (MFS) was determined as the interval between initial diagnosis and detection of the first metastasis.

## SUPPLEMENTARY MATERIALS FIGURES AND TABLES


