# Supplementary material for: Involvement of the FOXO6 transcriptional factor in breast carcinogenesis
Source: Oncotarget. 2017 Dec 30;9(7):7464–75. doi: 10.18632/oncotarget.23779 (PMC5800916; doi:10.18632/oncotarget.23779)
Supplement: Supplementary file 1 [file oncotarget-09-7464-s001.pdf]

Involvement of the FOXO6 transcriptional factor in breast carcinogenesis

SUPPLEMENTARY MATERIALS

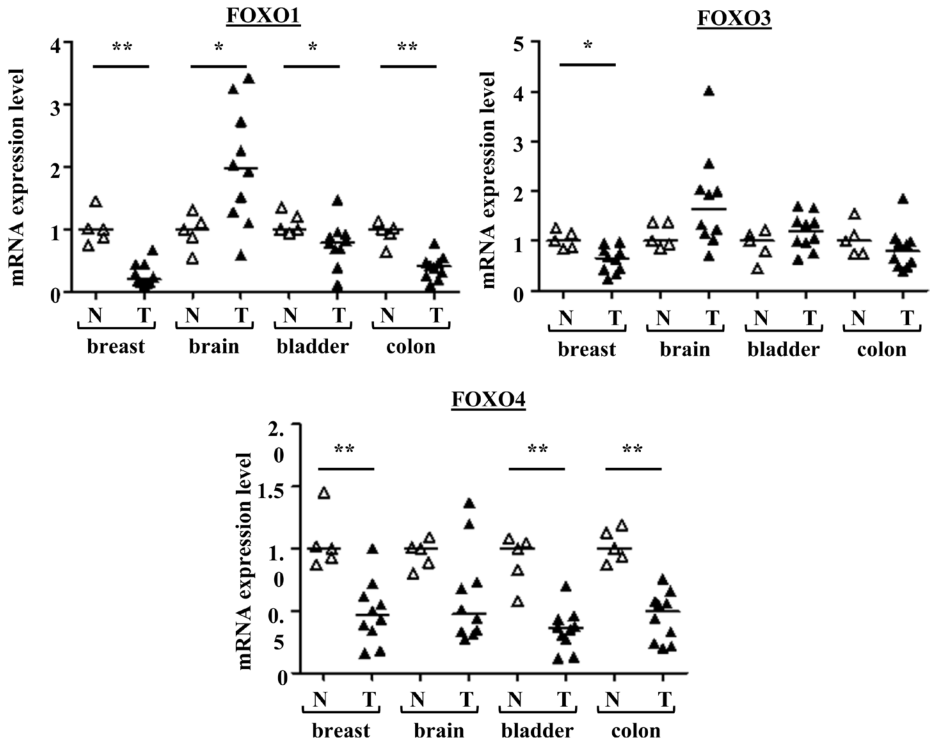

Supplementary Figure 1: *FOXO1*, *FOXO3*, and *FOXO4* mRNA expressions in various cancers and normal tissues. Scatter dot plot with median of qRT-PCR data for *FOXO1*, *FOXO3*, and *FOXO4* in the series of breast, brain, bladder and colon tissues (n = 5 normal tissues and n = 10 tumour tissues for each). p-values (Mann-Whitney U Test) are indicated: *, 0.01< p-value < 0.05; $^{**}p$-value < 0.01.

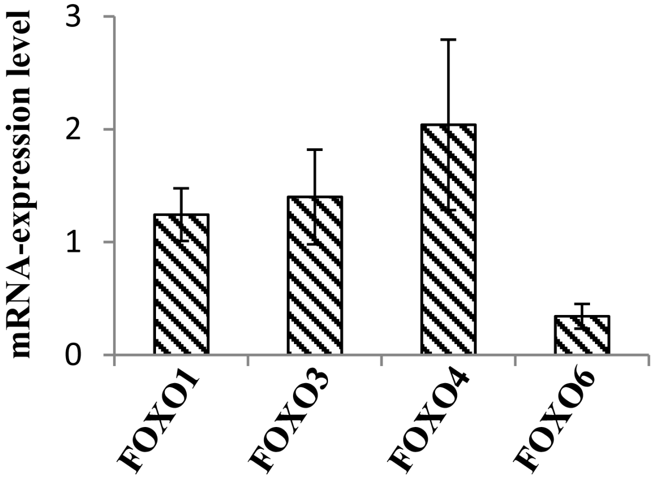

Supplementary Figure 2: Evaluation of the specificity of *FOXO6* siRNA. MDA-MB-468 cells were transfected with control siRNA or *FOXO6* siRNA. Six days after transfection, total mRNA was extracted and *FOXO1*, *3*, *4* and *6* expression levels were determined by qRT-PCR. For each FOXO, the mRNA expression detected in cells transfected with *FOXO6* siRNA was normalised to that detected in cells transfected with control siRNA. Results are expressed as mean +/– SD of three independent experiments.

Supplementary Table 1: mRNA expression of *FOXO* genes in different human normal tissues

Tissue	FOXO1	FOXO3	FOXO4	FOXO6
Breast	1938	442	1697	77
Ovary	**4162**	522	**4891**	132
Brain (whole)	450	276	2114	91
Brain cerebellum	284	726	1747	19
Small Intestine	1152	402	1188	59
Thyroid	2264	390	919	259
Heart	2190	507	1524	99
kidney	1485	460	1336	47
Liver	2032	400	1279	13
Lung	3176	723	2358	155
Trachea	2316	567	1004	93
Colon	1406	320	953	36
Bone marrow	872	**906**	2774	16
Spleen	2197	471	1240	72
Thymus	1480	424	1001	149
Skeletal Muscle	**4273**	**906**	**4669**	149
Testis	200	67	309	18
Uterus	**5060**	552	1170	119
Adrenal Gland	2276	302	**6818**	85
Salivary Gland	1105	373	1845	117
Spinal Cord	2068	519	1748	81
Fetal brain	409	774	399	**502**
Fetal liver	2394	623	3220	9
Placenta	517	224	**8828**	109
**Mediane**	1938	442	1697	91

Supplementary Table 2: mRNA expression of *FOXO* genes in breast cell lines

Cell Line	status	FOXO1	FOXO3	FOXO4	FOXO6
hTert HME1	N	0.35	1.55	0.18	0.39
MCF-12A	N	1.55	1.33	0.60	3.16
MCF-12F	N	2.00	0.76	1.78	3.02
HMEC	N	0.53	1.00	1.49	0.89
MCF-10-2A	N	0.61	0.62	1.12	3.43
MCF-10A	N	1.00	0.64	1.00	1.00
184B5	N	1.65	1.35	0.63	0.79
HCC-1428	T	0.23	0.87	1.67	**54.92**
BT483	T	0.27	1.55	1.14	**34.02**
MDA-MB-361	T	0.14	1.59	0.58	12.39
HCC-202	T	0.65	2.12	2.55	**40.16**
HCC-1187	T	1.18	1.19	0.92	15.12
T47D	T	0.17	0.78	0.60	8.38
MDA-MB-134-VI	T	0.08	0.46	1.06	28.65
PMC42	T	1.31	1.83	0.34	11.64
MCF7	T	0.23	1.38	0.35	11.72
BT474	T	0.18	2.81	0.64	21.59
SKBR3	T	0.56	0.45	0.32	29.38
CAMA1	T	0.24	0.81	0.70	1.95
MDA-MB-231	T	0.06	0.22	0.16	6.01
ZR75-1	T	0.08	0.54	0.49	14.31
HCC-1569	T	0.19	1.20	0.68	4.56
HCC-1954	T	0.89	0.99	0.32	22.12
MDA-MB-453	T	0.14	0.90	0.98	22.78
MDA-MB-415	T	0.21	2.22	4.13	**91,66**
HCC-1599	T	0.06	0.51	0.32	5.02
HCC-38	T	0.29	4.75	1.49	**93.90**
MDA-MB-436	T	0.24	1.09	0.11	5.41
MDA-MB-468	T	0.11	1.26	0.49	**35.32**
Hs 578T	T	0.35	1.23	0.68	2.82
HCC-1143	T	0.23	1.00	0.51	3.60
HCC-70	T	1.34	1.61	1.42	**39.04**
BT549	T	0.13	2.53	0.26	0.73
BT20	T	0.80	3.40	0.41	**32.53**
MDA-MB-157	T	0.19	0.67	0.14	5.27
HCC-1937	T	1.14	2.16	0.42	17.29
HBL100	T	0.09	0.93	0.26	4.94
HCC1500	T	0.00	0.39	0.92	27.02
MDA-MB435S	T	0.24	1.19	1.55	0.18

N: normal breast cell lines; T: tumorigenic breast cell lines; in bold: overexpression (defined as Ct values under 30 (values above 32 ($2^{\Delta Ct} = 2^{35-30} = 32$)).

Supplementary Table 3: Characteristics of the 527 breast tumors

Clinical biological parameters	Number of patients (%)	Number of patients with metastases (%)	*p*-value[a]
Total	527 (100)	210 (39.8)	
Age			
≤50	125 (23.7)	52 (41.6)	0.52 (NS)
>50	402 (76.3)	158 (39.3)	
SBR histological grade[b,c]			
I	60 (11.7)	12 (20.0)	**0.0019**
II	241 (47.1)	100 (41.5)	
III	211 (41.2)	94 (44.5)	
Lymph node status[d]			
0	159 (30.5)	48 (30.2)	**<0.0000001**
1–3	250 (47.9)	88 (35.2)	
>3	113 (21.6)	72 (63.7)	
Macroscopic tumor size[e]			
≤25 mm	248 (48.0)	77 (31.0)	**0.000015**
>25 mm	269 (52.0)	132 (49.1)	
ERα status			
Negative	181 (34.3)	76 (42.0)	0.10 (NS)
Positive	346 (65.7)	134 (38.7)	
PR status			
Negative	255 (48.4)	110 (43.1)	**0.025**
Positive	272 (51.6)	100 (36.8)	
ERBB2 status			
Negative	397 (75.3)	153 (38.5)	0.17 (NS)
Positive	130 (24.7)	57 (43.8)	
Molecular subtypes			
RH– ERBB2–	102 (19.4)	38 (37.3)	0.054 (NS)
RH– ERBB2+	72 (13.7)	36 (50.0)	
RH+ ERBB2–	295 (56.0)	115 (39.0)	
RH+ ERBB2+	58 (11.0)	21 (36.2)	
Histological types[f]			
Ductal	398 (89.6)	156 (39.2)	1.00 (NS)
Lobular	28 (6.3)	11 (39.3)	
Other	18 (4.1)	7 (38.9)	
PIK3CA mutation status[h]			
wild type	299 (67.0)	123 (20.5)	0.065 (NS)
mutated	147 (33.0)	50 (34.0)	

a: Log-rank test; NS: not significant; b: Scarff Bloom Richardson classification; c: informations available for 512 patients; d: informations available for 522 patients; e: informations available for 517 patients; f: informations available for 444 patients; h: informations available for 446 patients.

Supplementary Table 4: Primer sequences used for qRT-PCR

Gene	Upper primer (5′ to 3′)	Lower primer (5′ to 3′)
TBP	TGCACAGGAGCCAAGAGTGAA	CACATCACAGCTCCCCACCA
FOXO1	GTCAAGAGCGTGCCCTACTTCA	TGAACTTGCTGTGTAGGGACAGATTAT
FOXO3	AGATCTACGAGTGGATGGTGCGTT	AATCGACTATGCAGTGACAGGTTGTG
FOXO4	TGGTCCGTACTGTACCCTACTTCA	GGCGGATCGAGTTCTTCCAT
FOXO6	CCTGCGCATCAAGGGCAAG	GCACTCGGGGAGCTGTCGTC
